# Multi-channel anomaly detection using graphical models

**DOI:** 10.1007/s10845-024-02447-7

**Published:** 2024-07-13

**Authors:** Bernadin Namoano, Christina Latsou, John Ahmet Erkoyuncu

**Affiliations:** Centre of Digital Engineering and Manufacturing, Cranfield University, College Rd, Wharley End, Bedford, MK43 0AL UK

**Keywords:** Time-series, Anomaly detection, Multi-channel, Multivariate, Graphical model

## Abstract

Anomaly detection in multivariate time-series data is critical for monitoring asset conditions, enabling prompt fault detection and diagnosis to mitigate damage, reduce downtime and enhance safety. Existing literature predominately emphasises temporal dependencies in single-channel data, often overlooking interrelations between features in multivariate time-series data and across multiple channels. This paper introduces G-BOCPD, a novel graphical model-based annotation method designed to automatically detect anomalies in multi-channel multivariate time-series data. To address internal and external dependencies, G-BOCPD proposes a hybridisation of the graphical lasso and expectation maximisation algorithms. This approach detects anomalies in multi-channel multivariate time-series by identifying segments with diverse behaviours and patterns, which are then annotated to highlight variations. The method alternates between estimating the concentration matrix, which represents dependencies between variables, using the graphical lasso algorithm, and annotating segments through a minimal path clustering method for a comprehensive understanding of variations. To demonstrate its effectiveness, G-BOCPD is applied to multichannel time-series obtained from: (i) Diesel Multiple Unit train engines exhibiting faulty behaviours; and (ii) a group of train doors at various degradation stages. Empirical evidence highlights G-BOCPD's superior performance compared to previous approaches in terms of precision, recall and F1-score.

## Introduction

Time-series data analysis, the process of extracting meaningful knowledge from time-ordered data and using this knowledge to diagnose and predict the past and future behaviour of assets, has become a field of interest for both academia and industry in various domains, including transportation, medicine and economics (Choi et al., 2021). With the evolution of Industry 4.0 and the vast amount of available data, time-series analysis has attracted a lot of interest in maintenance systems to detect or forecast asset health status, prevent potential catastrophic failures, allow for better maintenance planning and, in turn, reduce costs (Manco et al., 2017; Zhao et al., 2020). Time-series data can be described as a collection of observations arranged in a time order (Hamilton, 2020). Classification, clustering, anomaly (or outlier) detection and forecasting are the most common data mining techniques employed in the current literature for time-series data analysis (Tang et al., 2019). In this context, anomaly detection refers to the process of identifying anomalous data with different severity characteristics such as fluctuation (i.e. statistical outliers) that do not conform to the common rules in time-series. An anomaly in time-series refers to a data point or sequence that deviates from the normal range. By learning the normal system operations patterns and identifying the outliers, system failures can be promptly detected.

Anomalies can be effectively detected if the properties of time-series data, including temporality, dimensionality and non-stationarity, and the status of data quality are properly understood. A detailed explanation of the properties can be found in the work conducted by Choi et al. (2021). Traditional anomaly detection approaches, including linear-based, distance-based and density-based (Alqahtani et al., 2021) are limited to manipulating univariate or single-channel time-series data sets (i.e. capturing anomalies for a single feature or variable), while they are not sufficiently advanced to handle the absence of labelled training data (Zhao et al., 2020). Additionally, as the complexity of industrial applications increases and multi-channel multivariate time-series data becomes more prevalent, the aforementioned anomaly detection methods have become impractical. In this work, multi-channel,^1^ multivariate,^2^ nonstationary^3^ and noisy^4^ time-series data will be studied. A typical example of multi-channel multivariate time-series data can be considered the data generated by sensors attached to railway train engines. Thus, on Diesel Multiple Unit (DMU) train engines, a single channel time-series data is considered the data collected from one engine. The channels should have a similar data architecture. Each channel comprises multivariate sensor measurement data (e.g. volume, pressure, temperature, speed and control signals).

With the increased availability of data, data-driven methods effectively utilise historical data to learn asset behaviour and classify or cluster newly observed data. Recent literature has explored approaches, including support vector machine methods for induction motors (Gangsar & Tiwari, 2019), neural network-based applications for engineering anomaly detection (Lin et al., 2019), deep convolutional neural networks (DCNN) for track damage detection (Chen et al., 2019; James et al. 2019; Yuan et al., 2020) and back-propagation neural networks for track spikes detection (Lu & Shen, 2020), among others. Additionally, applying fuzzy C-means clustering methods, Yin and Huang (2015) addressed faults in railway vehicle suspension. Similarly, clustering methods, such as dynamic time warping-based approaches, have been investigated for fault detection in railway point machine (Du et al., 2019). Other common approaches include shapelet analysis (Beggel et al., 2018) and matrix profile methods (Yeh et al., 2018) for unified motif discovery, anomaly detection and fault diagnosis. While these studies offer valuable insights into potential methods, their focus remains on single-variate time-series, limiting their scope.

In the context of anomaly detection and diagnosis for multivariate time-series data, relevant works are discussed. Xi et al. (2006) proposed numerosity reduction to enhance the efficiency of Dynamic Time Warping (DTW) in one-nearest-neighbour for time-series classification. In 2013, Senin and Malinchik introduced SAX-VSM, combining Symbolic Aggregate Approximation and Vector Space Model for automated discovery and ranking of interpretable characteristic patterns, achieving accurate classification. Patri et al. (2014) proposed a shapelet forest approach for multivariate times series classification, featuring shapelet extraction, distance calculation, and new feature creation for fault detection. In 2017, Lee et al. presented a CNN model for fault detection and classification in semiconductor manufacturing processes. In more recent literature, Hsu and Liu (2021) introduced a CNN model employing data augmentation and a diagnostic layer for adaptive feature extraction, fault detection and diagnosis in semiconductor manufacturing. Chen et al. (2023) proposed LR-SemiVAE, a long-short term memory (LSTM)-based semi-supervised variational autoencoder (VAE) for anomaly detection, and RT-SemiVAE, a transformer-based VAE with parallel multihead attention, offering more accurate anomaly detection and precise root cause localisation in a large-scale cloud environment. Additionally, Kim et al. (2023) introduced an unsupervised time-series anomaly detection method using Transformers, outperforming LSTM and CNN with self-attention mechanisms. In 2024, Bignoumba et al. presented ALignment-driven Neural Network, transforming data into pseudo-aligned latent values through a novel matrix representation. Although these methods have been applied with varying degrees of success, their application to real-world operational data, with multiple channels of data and diverse complexities, remains limited.

In the context of anomaly detection and diagnosis for multi-channel time-series data, relevant works are discussed. Zheng et al. (2014) proposed a multi-channel DCNN to handle varying lengths of sensor data. The CNN initially learns features from each sensor's subsequences using one channel, enabling the entire model to understand the features of different sensors. The CNN-extracted features are then combined and utilised with a conventional Multi-Layer Perceptron for time-series classification. An MC-CNN was also developed by Yang et al. (2015) to identify human movements, learning features and classifying datasets. Moreover, Canizo et al. (2019) proposed a deep learning-based method for supervised multi-channel time-series anomaly detection in industrial settings, utilising CNNs and Recurrent Neural Networks using independent CNNs (convolutional heads) for each sensor. Similarly, Huang et al. (2020) adopted multi-domain features into a DCNN model for tool wear prediction. In recent literature, Xu et al. (2023) presented a model for multivariate time-series anomaly detection, employing a dual-channel feature extraction module that focuses on spatial and time features. Although various approaches have been proposed for either multi-channel or multivariate data, there is a lack of methods that effectively handle anomalies in both types of time-series simultaneously.

In multi-channel multivariate time-series anomaly detection, capturing both temporal dependency (i.e. relations between current and previous states) and interrelations between features (i.e. intra-dependency) is crucial. While intra-dependency is not a new research topic, capturing the interrelations among multiple channels has not yet been addressed (Li et al., 2022). Existing literature suggests that interrelations can be extracted from feature sparsity and effectively represented through graphical models (Fang et al. 2023; Xu et al., 2023). Graphs can visually represent time-series data, with nodes denoting process variables at each timestamp and edges representing connections between nodes. They can effectively model dependencies in multi-channel multivariate time-series data, capturing topological structures and causal relationships between features ( Chen et al., 2022; Deng & Hooi, 2021; Zhao et al., 2020). In recent years, there has been an increasing interest in the use of graph machine learning for anomaly detection and diagnosis. Notably, Wu et al. (2020) introduced a graph learning approach for multivariate time-series data, able to extract the directed spatial dependency between features. Additionally, Tao and Du (2023) proposed a graph-based sparse learning framework for anomaly detection on unstructured point cloud data, representing smooth free-form surfaces. The literature also highlights relevant research on graph convolutional networks (GCN), graph dynamic autoencoders and fault detection algorithms that integrate graph modelling and minimum spanning tree graphs (Goswami et al., 2023). Motivated by the success of graphical models in diagnosing anomalies in industrial systems, this study introduces a method based on a graphical model for multi-channel multivariate time-series. The proposed model can capture inter-dependency within single-channel data and intra-dependency among multiple channels. By introducing multi-channel feature extraction, the learning process of the graphical model can capture the correlation between the hidden features across different channels, enabling prompt, accurate and automated anomaly detection and diagnosis.

This research work contributes to the literature by proposing a generic graphical model-based annotation method for detecting anomalies in multiple channels of sensor data with a shared structure in an automatic way. By adopting the graphical lasso algorithm method for a dependant graphical-based model, the proposed solution can capture the conditional dependency of channel features and dynamically detect anomalies based on unlabelled multi-channel data establishing relationships between multiple features at every time step. Therefore, the contributions of this work are summarised as follows:

- A novel graphical model-based annotation method, called G-BOCPD, is introduced for detecting anomalies in multi-channel multivariate time-series data automatically.
- A hybridisation of the graphical lasso and expectation maximisation algorithms is proposed for capturing the internal and external dependencies in multi-channel multivariate time-series data. The segments are annotated to highlight the difference in behaviour within the multivariate time-series. G-BOCPD model computes alternatively the concentration matrix and annotation of the segments, employing the graphical lasso and a minimal path clustering method, respectively.
- G-BOCPD method is validated using two real-world engineering datasets: (i) DMU train engines with faulty behaviours; and (ii) a group of train doors data with different degradation stages. Moreover, quantitative experiments are conducted to demonstrate that the proposed method can provide competitive results compared to alternative methods in the field in terms of precision, recall and F1-score.

The remainder of the paper is organised as follows: Section II describes the methodological approach used for developing the graphical model-based annotation method, G-BOCPD, for anomaly detection in multi-channel multivariate time-series. Section III presents the problem motivation for the proposed method, including a real-world multi-channel multivariate time-series data problem. Additionally, the proposed G-BOCPD is validated through a case study in anomaly detection of multiple unit diesel engines. Section IV presents a summary of critical discussion on the proposed method while comparing the results of this work with those obtained in the literature on methods for detecting anomalies in multivariate time-series. Finally, the conclusions and future work are highlighted in Section V.

## Methodology

**Fig. 1:**
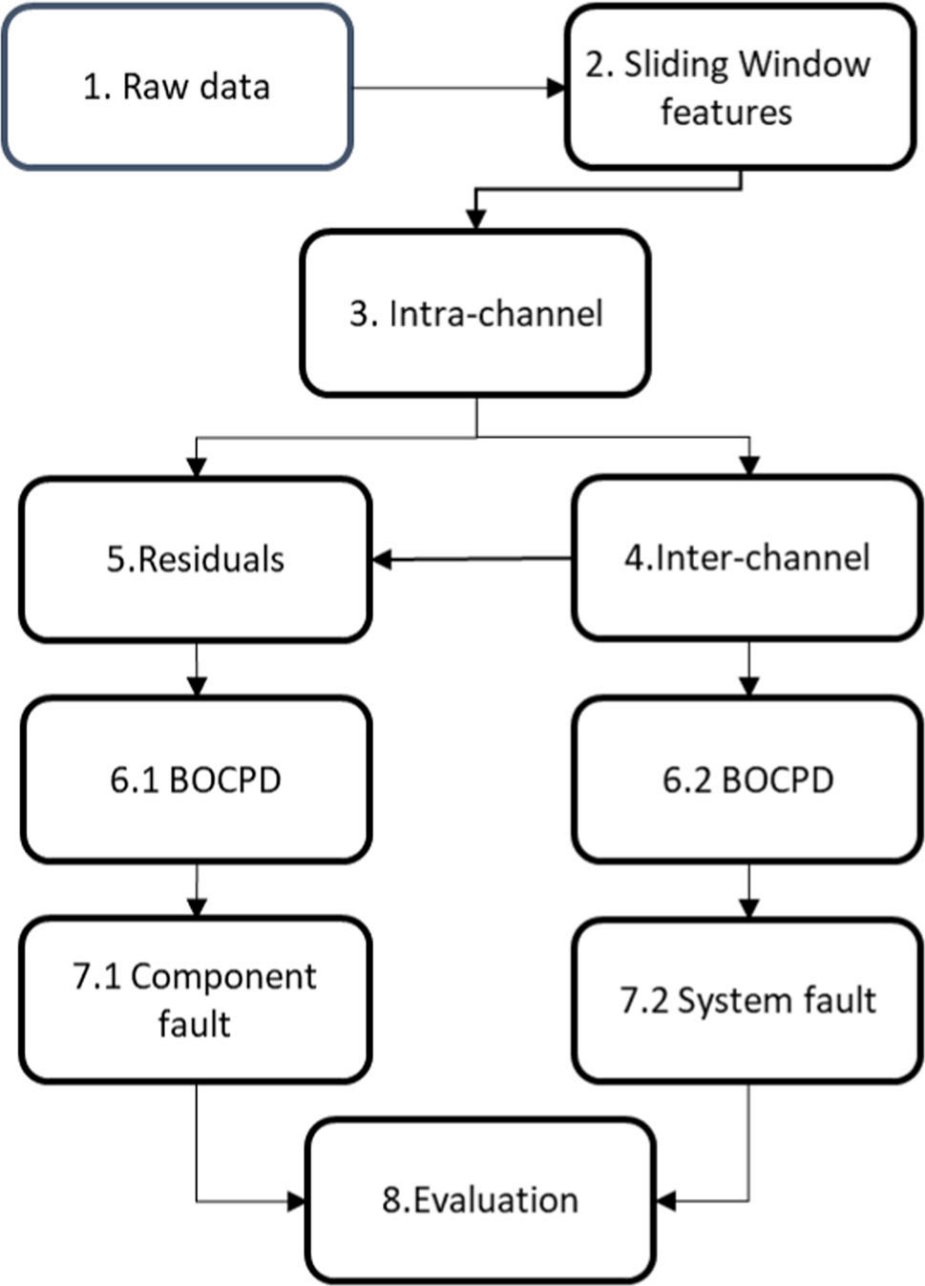
Methodological approach

The methodology employed for the anomaly detection is shown in Fig. 1. The step 1, where raw data are collected and organised into multiple channels. Step 2 involves implementing a sliding window technique in order to extract relevant time indexed features that may be sensitive to anomaly conditions. Step 3 estimate intra-channel dependency estimation, i.e., make use of graphical models to estimate the conditional dependencies within individual channels of the dataset. Step 4 pertains to inter-channel dependency estimation, also using graphical models to ascertain the dependencies and relationships between different channels. In step 5, the focus shifts to the computation of residuals, which detect intra channel anomalies. The residuals represent each channel specifics variations. Steps 6.1 and 6.2 both involve Bayesian Online Change Point Detection, first applied to each channel to detect change points in 6.1, and to detect change at inter-channel level 6.2 for system-wide fault detection. Steps 7.1 and 7.2 build upon the results of the BOCPD, with 7.1 addressing whether or not change point detected are component faults in steps 6.1, and 7.2 focusing on inter-channel faults, which involve the assessment of identified changes detected in step 6.2. Finally, Step 8 encompasses the evaluation phase, where the results of the previous steps are assessed, using performance metrics to ensure the efficacy and accuracy of the methodology.

### Problem definition

Given a multidimensional time-series, ${\varvec{T}}=\{{{\varvec{T}}}_{1},{{\varvec{T}}}_{2},\dots {{\varvec{T}}}_{{\varvec{k}}},..{{\varvec{T}}}_{{\varvec{K}}}\}$, where each ***T***_***k***_ $\in {R}^{np}$ a multivariate time-series and ${{\varvec{T}}}_{{\varvec{k}},{\varvec{i}}}$ the observation at the time index *i* of the time-series ***T***_***k***_. ${{\varvec{T}}}_{{\varvec{k}},{\varvec{i}}}$ is a vector of $p$ variables entries values. To examine the dependency between the multivariate time-series, two latent variables ${\varvec{C}}$ and ${\varvec{X}}$ are introduced (Xie et al., 2016) such that:

1 $${\varvec{T}}_{\varvec{k}}={\varvec{C}}+{\varvec{X}}_{\varvec{k}}$$

where ***C*** captures shared behaviour among all the multivariate time-series and ${{\varvec{X}}}_{{\varvec{k}}}$ the variation within the specific multivariate time-series ${{\varvec{T}}}_{{\varvec{k}}}$. In some conditions, ***C*** represents the behaviour shared across channels whereas ${{\varvec{X}}}_{{\varvec{k}}}$ can be seen as a local behaviour of the component. When all the time-series have the same behaviour, the ${{\varvec{X}}}_{{\varvec{k}}}$ represents anomalous behaviour, or noise measurement as it captures the deviation of the local component from the common behaviour. The present work assumes that latent variables ***C*** and $\left\{{{\varvec{X}}}_{{\varvec{k}}}\right\}, k = 1, 2, \dots , K$ are mutually independent and follow a Gaussian distribution. It has been shown in such settings (Xie et al., 2016) that:

2 $$var\left({\varvec{C}}\right)=cov({{\varvec{T}}}_{{\varvec{k}}},\boldsymbol{ }{{\varvec{T}}}_{{\varvec{l}}}) k \ne l$$

Considering ${\boldsymbol{\Theta }}_{{\varvec{s}}}$ the concentration matrix of the window ${{\varvec{w}}}_{{\varvec{s}}}$, the inter-channel layer aims to estimate ${\boldsymbol{\Theta }}_{{\varvec{s}}}$ for all the *K* multivariate time-series and ${\boldsymbol{\Theta }}_{0,{\varvec{s}}}$ such that:

3 $$var\left({\mathbf{C}}_{\mathbf{s}}\right)={\boldsymbol{ }\boldsymbol{\Theta }}_{0,{\varvec{s}}}^{-1}$$

At the system layer, the estimation of the concentration matrix ${\boldsymbol{\Theta }}_{{{\varvec{T}}}_{{\varvec{k}}}}$ is as follows:

4 $${\boldsymbol{\Theta }}_{{{\varvec{T}}}_{{\varvec{k}}},{\varvec{s}}}={({\boldsymbol{ }\boldsymbol{\Theta }}_{0,{\varvec{s}}}^{-1}+{\boldsymbol{ }\boldsymbol{\Theta }}_{{\varvec{k}},{\varvec{s}}}^{-1})}^{-1}$$

In these settings, the following likelihood is estimated:

5 $$\mathop {{\text{argmin}}}\limits_{\varvec{\Theta}}  \sum\limits_{k = 1}^k {\left[ {\overbrace {||\lambda *{{\varvec{\Theta}} _{\varvec{0,s}}}|{|_1}}^{sparsity} + \overbrace { - LL\left( {\varvec{T_k},\varvec{{\Theta _{k,s}}}} \right)}^{\log likelihood}} \right]}$$

with $\lambda$ representing a non-negative tuning parameter guaranteeing the sparsity. A large $\lambda$ value, for instance, makes the entries of the concentration matrix converge to zeros, ${||\bullet ||}_{1}$ is the L_1_-norm, $*$, the element-wise matrix product is also known as the Hadamard product.

6 $$ \begin{aligned} & LL\left({\varvec{T}},{\boldsymbol{\Theta }}_{{\varvec{u}},{\varvec{s}}}\right)\\ & \quad=-\frac{1}{2}\left[\left({\varvec{T}}- {{\varvec{\mu}}}_{{\varvec{u}},{\varvec{s}}}\right){\boldsymbol{\Theta }}_{{\varvec{u}},{\varvec{s}}}\left({\varvec{T}}- {{\varvec{\mu}}}_{{\varvec{u}},{\varvec{s}}}\right)+ \text{log} det({\boldsymbol{\Theta }}_{{\varvec{u}},{\varvec{s}}})+\gamma \right] \end{aligned}$$

${{\varvec{\mu}}}_{{\varvec{s}}}$ is the empirical mean of the samples to the window ${{\varvec{w}}}_{{\varvec{s}}}$, $\gamma$ a constant value, $det({\boldsymbol{\Theta }}_{{\varvec{u}},{\varvec{s}}})$ is the determinant of the matrix ${{\varvec{\Theta}}}_{{\varvec{u}},{\varvec{s}}}$.

A sub-problem of optimisation (5) has been investigated in (Hallac et al., 2018) for only one multivariate time-series (*K* = *1*). A similar resolution approach is taken here, as the optimisation problem shares the same characteristics, i.e., it is a highly non-convex mixed discrete and continuous optimisation problem, with $\boldsymbol{\Theta }$ being the sets of the precision. The continuous optimisation problem is solved using the ADMM (Alternating Direction Method of Multipliers) algorithm.

### Problem resolution

A closed form of the continuous optimisation for each multivariate time-series ${\text{T}}_{k}$ in (5) can be rewritten as follow:

7 $$\underset{\varvec{\Theta }}{\text{max}}\frac{1}{2}\sum_{s=1}^{S}\left(\text{log}{det}{\varvec{\Theta }}_{{\varvec{k}},{\varvec{s}}}-\text{ tr}\left({{\varvec{R}}}_{{\varvec{k}},{\varvec{s}}}{\varvec{\Theta }}_{{{\varvec{T}}}_{{\varvec{k}}},{\varvec{s}}}\right)-\uplambda {\left|\left|{{\varvec{Z}}}_{{\varvec{k}},{\varvec{s}}}\right|\right|}_{1}\right)$$

Subject to ${\boldsymbol{\Theta }}_{{\varvec{k}},{\varvec{s}}}$ positive and ${{\varvec{Z}}}_{{\varvec{k}},{\varvec{s}}}={\boldsymbol{\Theta }}_{{\varvec{k}},{\varvec{s}}}$

$\text{tr}({\varvec{R}}\boldsymbol{\Theta })$ is the trace of the matrix ${\varvec{R}}\boldsymbol{\Theta }$, ***R*** the empirical covariance matrix and $\boldsymbol{\Theta }=\left\{{\boldsymbol{\Theta }}_{{\varvec{k}},{\varvec{s}}}\right\},\text{s}=1..\text{S}$. ${\varvec{Z}}$ a consensus variable (Danaher et al., 2014). The augmented Lagrangian multipliers scaled by Frobenius norm of the minimisation form of the problem (8) defined by (Boyd, 2010) is given by:

8 $$ \begin{aligned} & {L}_{\rho }\left({\varvec{\Theta }}_{{\varvec{k}},{\varvec{s}}},{{\varvec{Z}}}_{{\varvec{k}},{\varvec{s}}},{{\varvec{U}}}_{{\varvec{k}},{\varvec{s}}}\right)\\ &\quad= \left\{-\text{log}{det}{\varvec{\Theta }}_{{\varvec{k}},{\varvec{s}}}+\text{ tr}\left({{\varvec{S}}}_{{\varvec{k}},{\varvec{s}}}{\boldsymbol{\Theta }}_{{\varvec{k}},{\varvec{s}}}\right)+\uplambda {\left|\left|{{\varvec{Z}}}_{{\varvec{k}},{\varvec{s}}}\right|\right|}_{1}\right\}\\ &\quad+\frac{\rho }{2} {\left|\left|{\varvec{\Theta }}_{{\varvec{k}},{\varvec{s}}}-{{\varvec{Z}}}_{{\varvec{k}},{\varvec{s}}}+{{\varvec{U}}}_{{\varvec{k}},{\varvec{s}}}\right|\right|}_{F}- \frac{\rho }{2}{\left|{|{\varvec{U}}}_{{\varvec{k}},{\varvec{s}}}|\right|}_{F}^{2} \end{aligned}$$

where $\rho >0$ is the ADMM penalty parameter, ${{\varvec{U}}}_{{\varvec{k}},{\varvec{s}}}$ are dual variables. ${\boldsymbol{\Theta }}_{{\varvec{k}}}=\{{\boldsymbol{\Theta }}_{{\varvec{k}},{\varvec{s}}}\}$, ${{\varvec{Z}}}_{{\varvec{k}}}=\{{{\varvec{Z}}}_{{\varvec{k}},{\varvec{s}}}\}$,${{\varvec{U}}}_{{\varvec{k}}}=\{{{\varvec{U}}}_{{\varvec{k}},{\varvec{s}}}\}$, s = 1..*S*, ${\left||\bullet |\right|}_{F}^{2}$ is the Frobenius norm. The optimal solution to the problem (9) provides an estimation of the concentration matrix ${\boldsymbol{\Theta }}_{{\varvec{k}},{\varvec{s}}}$’s. ADMM for multivariate works in three main steps as follows:

At the ith iteration, update the parameter ${\boldsymbol{\Theta }}_{{\varvec{k}}},{{\varvec{Z}}}_{{\varvec{k}}},{{\varvec{U}}}_{{\varvec{k}}}$:

- $${\varvec{\Theta}}-\mathbf{U}\mathbf{p}\mathbf{d}\mathbf{a}\mathbf{t}\mathbf{e}: {{\boldsymbol{\Theta }}_{{\varvec{k}}}}^{(i)}=\underset{\Theta }{\text{argmin}}\left\{{L}_{\rho }\left(\boldsymbol{\Theta },{{{\varvec{Z}}}_{{\varvec{k}}}}^{(i-1)},{{{\varvec{U}}}_{{\varvec{k}}}}^{(i-1)}\right)\right\}$$
- $${{\mathbf{Z}-\mathbf{U}\mathbf{p}\mathbf{d}\mathbf{a}\mathbf{t}\mathbf{e}: {\varvec{Z}}}_{{\varvec{k}}}}^{(i)}=\underset{\text{z}}{\text{ argmin}}\left\{{L}_{\rho }\left({{\boldsymbol{\Theta }}_{{\varvec{k}}}}^{(i)},{{\varvec{Z}}}_{{\varvec{k}}},{{{\varvec{U}}}_{{\varvec{k}}}}^{(i-1)}\right)\right\}$$
- $$\mathbf{U}-\mathbf{U}\mathbf{p}\mathbf{d}\mathbf{a}\mathbf{t}\mathbf{e}:$$
  $${{{\varvec{U}}}_{{\varvec{k}}}}^{(i)}={{{\varvec{U}}}_{{\varvec{k}}}}^{(i-1)}+({{\boldsymbol{\Theta }}_{{\varvec{k}}}}^{\left(i\right)}-{{{\varvec{Z}}}_{{\varvec{k}}}}^{(i)})$$

The general algorithm of the multivariate ${\text{Z}}_{k}$ is presented below:

- For each $${{\varvec{T}}}_{{\varvec{k}}} k=1..K$$
- Initialise the variables: ${\boldsymbol{\Theta }}_{{\varvec{k}},{\varvec{s}}}=\text{I}, {{\varvec{Z}}}_{{\varvec{k}}}=0, and {{\varvec{U}}}_{{\varvec{k}},{\varvec{s}}}=0 for each s=1..S$
- Initialise $$\rho >0$$

For each $$i=1..\text{ until convergence}$$

(i) ${\varvec{\Theta}}-\mathbf{U}\mathbf{p}\mathbf{d}\mathbf{a}\mathbf{t}\mathbf{e}: {{\boldsymbol{\Theta }}_{{\varvec{k}},{\varvec{s}}}}^{(i)}=\underset{{\varvec{\Theta}}}{\text{argmin}}\left\{{L}_{\rho }\left(\boldsymbol{\Theta },{{{\varvec{Z}}}_{{\varvec{k}},{\varvec{s}}}}^{(i-1)},{{{\varvec{U}}}_{{\varvec{k}},{\varvec{s}}}}^{(i-1)}\right)\right\}$ using eigen decomposition method proposed by (Witten and Tibshirani 2009).
(ii) $${{\mathbf{Z}-\mathbf{U}\mathbf{p}\mathbf{d}\mathbf{a}\mathbf{t}\mathbf{e}: {\varvec{Z}}}_{{{\varvec{T}}}_{{\varvec{k}}}}}^{(i)}=\underset{\text{z}}{\text{ argmin}}\Big\{{L}_{\rho }\Big({{\boldsymbol{\Theta }}_{{\varvec{k}},{\varvec{s}}}}^{(i)},\boldsymbol{ }{\varvec{Z}},{{\mathbf{U}}_{\mathbf{k},\mathbf{s}}}^{(i-1)}\Big)\Big\}$$
(iii) $$\mathbf{U}-\mathbf{U}\mathbf{p}\mathbf{d}\mathbf{a}\mathbf{t}\mathbf{e}:$$
  $${{{\varvec{U}}}_{{\varvec{k}},{\varvec{s}}}}^{(i)}={{{\varvec{U}}}_{{\varvec{k}},{\varvec{s}}}}^{(i-1)}+({{\boldsymbol{\Theta }}_{{\varvec{k}},{\varvec{s}}}}^{\left(i\right)}-{{{\varvec{Z}}}_{{\varvec{k}},{\varvec{s}}}}^{(i)})$$

In these settings, convergence is reached when ${{\boldsymbol{\Theta }}_{{\varvec{k}},{\varvec{s}}}}^{(i-1)}= {{\boldsymbol{\Theta }}_{{\varvec{k}},{\varvec{s}}}}^{(i)}$ to avoid convergence issues, maximum iteration is set as a parameter (set to 1000 for the experiment). The presented graphical lasso method above provides a concentration matrix for each multivariate segment. Concentration matrix of the system at the window ${{\varvec{w}}}_{{\varvec{s}}}$ is matrix ${\sum }_{{\varvec{s}}}$ where the diagonal represents components layer concentration matrices.

9 $${\sum }_{{\varvec{s}}}=\left(\begin{array}{ccc}{\boldsymbol{\Theta }}_{0,{\varvec{s}}}& \cdots & 0\\ \vdots & \ddots & \vdots \\ 0& \cdots & {\boldsymbol{\Theta }}_{{\varvec{k}},{\varvec{s}}}\end{array}\right)$$

So far, the estimation of the parameter concern only ${\boldsymbol{\Theta }}_{{\varvec{k}}}$’s. A natural way of ${\boldsymbol{\Theta }}_{0,{\varvec{s}}}$ is computing the ${\boldsymbol{\Theta }}_{0,{\varvec{s}}}^{-1}$’s representing the variance through a regression-based algorithm by using all pairs of variables of the observations. Xie et al. (2016) have shown that its estimation can be done for the observation belonging to the window ${{\varvec{w}}}_{{\varvec{s}}}$ using:

10 $$\dot{{\boldsymbol{\Theta }}_{0,{\varvec{s}}}^{-1}}= \frac{1}{K\left(K-1\right)N}\sum_{k=1}^{K}\sum_{i=1}^{n}{{\varvec{T}}}_{{\varvec{k}},{\varvec{i}}}\bullet {{{\varvec{T}}}_{{\varvec{k}},{\varvec{i}}}}^{T}$$

Therefore, the $\dot{{\boldsymbol{\Theta }}_{{\varvec{k}},{\varvec{s}}}}$ can be deduced from the Eq. (4):

11 $$\dot{{\boldsymbol{\Theta }}_{{\varvec{k}},{\varvec{s}}}}={\left({\boldsymbol{ }\boldsymbol{\Theta }}_{0,{\varvec{s}}}^{-1}-{\boldsymbol{ }\boldsymbol{\Theta }}_{{\varvec{k}},{\varvec{s}}}^{-1}\right)}^{-1} $$

To guarantee the positivity of ${\Theta }_{k,s}{^{\prime}}s$, semidefinite positiveness projection (Xu & Shao, 2012) can be used as follows:

12 $${\Theta }_{k,s}=\underset{\Theta }{\text{argmin}}{ \left|\left|{\varvec{\Theta}}-\dot{{\boldsymbol{\Theta }}_{{\varvec{k}},{\varvec{s}}}}\right|\right|}_{\infty } k=0..K$$

where ${\left|\left|\bullet \right|\right|}_{\infty }$ is the infinite norm.

### Tracking anomalies through changes

With the concentration matrixes ${\boldsymbol{\Theta }}_{0,{\varvec{s}}}^{-1}$ and ${\boldsymbol{\Theta }}_{{\varvec{k}},{\varvec{s}}}$ computed over the sliding window, a change point detection algorithm can be used for anomaly detection as ${\boldsymbol{\Theta }}_{{\varvec{k}},{\varvec{s}}}$ are stationary signals. Hence, BOCPD method (Namoano et al., 2020a, b, c) is used to detect anomalies at both channel and intra-channel levels.

## USE CASES

### Datasets

#### Engines

**Fig. 2:**
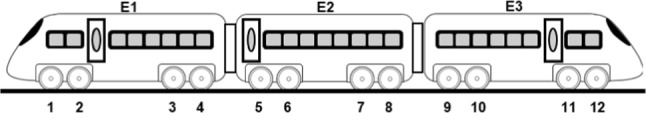
Train engine multiple units with three engines in the carriages E1, E2, and E3

The research reported in this paper is motivated by a real-world problem of anomaly detection on multiple system components. Figure 2 illustrates a diesel multiple-unit train, composed of three carriages. The engines share the load generated from the driver actions. In each engine, sensors, placed in various components (e.g. cooling, exhaust, shaft speed), continuously gather data such as the rotational frequency, charge air pressure, cooling temperature and pressure, as well as measurements of the exhaust gases and ambient temperature.

**Fig. 3:**
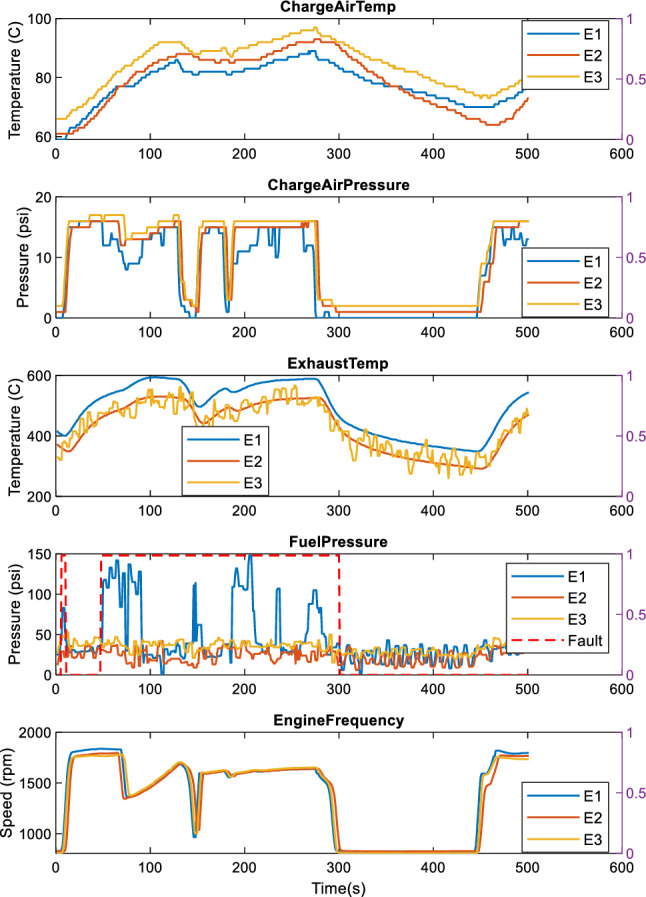
Engine normal operation modes with local faults of the fuel pressure in engine E1

**Fig. 4:**
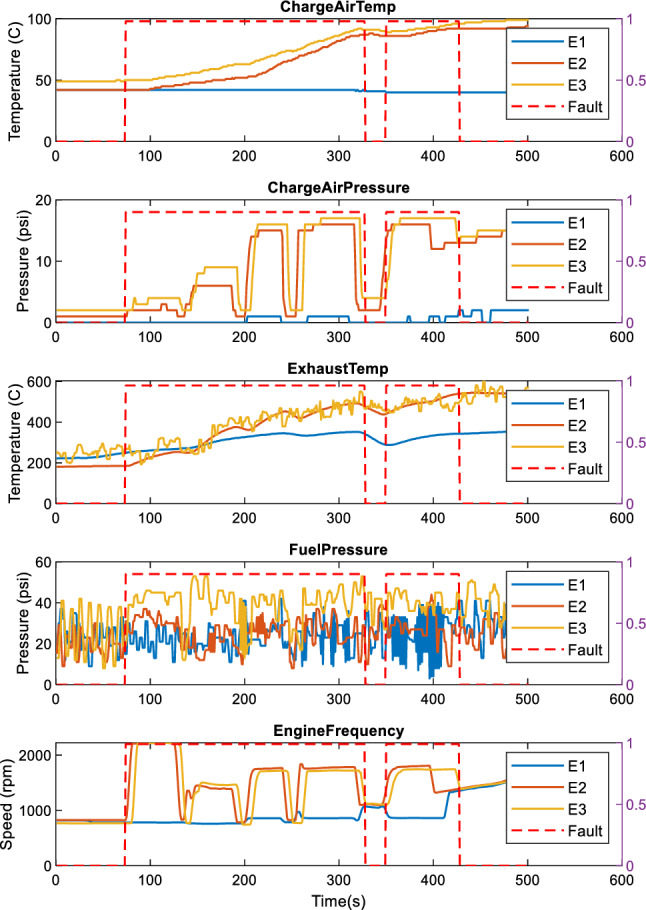
Engine normal operation modes with low power fault in engine E1

Figures 3 and 4 show examples of several signals overlayed, collected during the train operation. Patterns comprise idling mode where the engines are on (engine rotational speed is approximatively steady and around 800 rpm), acceleration in which there are rise in both pressure and temperature signals, gear changes, and deceleration where both pressures and temperature signals decrease, and the rotational speed changes to the idling rpm. The examples of signals shown include the engine charge air temperature (ChargeAirTemp), the charged air pressure (ChargeAirPressure), exhaust gas temperature (ExhaustTemp), internal fuel pressure (FuelTemp), and engine rotational speed. Each subplot represents the same signal for the three engines.

Figure 4 shows an example of a fuel pressure fault in engine E1. This pressure fault does not affect the E1 engine overall performance as the other signals in comparison to the other engines (the three engines produce roughly the same rotational speed). Conversely, in Fig. 4 where lower power event faults are observed, all the other signals of E1 are affected. Although Fig. 3 shows that anomaly has been detected only in engine E1, different faults have been observed in all the engines. It can be, thus, seen that there is a need to capture the relationship within one channel signal and between channel signals for the detection of faults occurring in the system.

Two types of datasets have been used for the analysis. The first category represents a working condition with white noise where due to some unknown cause, the charge air valve has unusual behaviour, leading to a shortage of air in one of the engines. In the second category, one of the engines often does not generate sufficient power to meet the demand, causing low-power events. The analysis aims to annotate the data such that clusters represent the normal conditions and faulty behaviour. The engine datasets are available on the Cranfield cord repository (Namoano et al., 2020b).

#### Doors

**Fig. 5:**
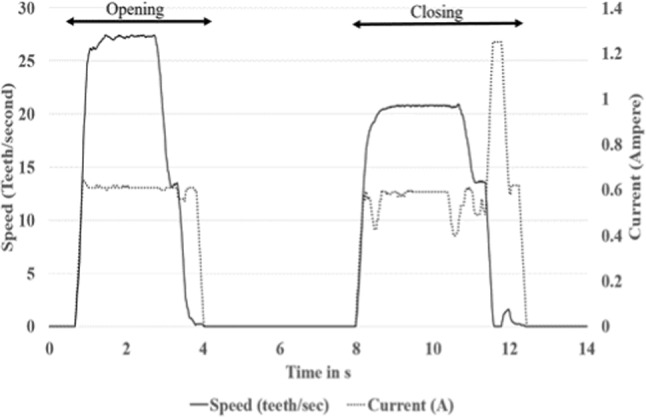
An example of a door opening and closing profile

The second dataset used is from doors and is collected at a frequency of 20 Hz, where many doors from the same manufacturers are supposed to have the same opening and closing profile. Datasets contain opening and closing speed and the current. As the rate of the use of these doors may not be the same depending on their location as well as the number of users, one may be interested in their variability instead of one component degradation. A way, therefore, to tackle this issue is through multivariate annotations. Figure 5 shows the opening and closing profiles, respectively. In the opening profile, the speed, as well as the current, increased steadily up to a maximum. A slight flat constant curve follows it and then decreases to zero. A dip can be seen during the decreasing phase, at roughly half the maximum value, due to a controller avoiding fast decrease, which could lead to severe damage to the door. As it reaches its maximum opening position, the door stays in the “open state” for several seconds before triggering the closing motion. The closing profile follows a similar pattern to the opening, with three main differences visible in the current motion:

- The peak in the closing profile is lower than the opening one, leading to a larger closing time window.
- Depending on the position of the door with the ground floor (tilted or flat ground), an additional gravitational speed may be added. A controller, therefore, adjusts the door speed by decreasing or increasing the current. Those can be seen in the current speed profile within the time range from 8 to 10 s.
- At the end of the closing profile, an abrupt change in the current can be seen, followed by a slight speed bump. This helps to push the door to its maximal reachable position, where a locking process can be triggered.

**Fig. 6:**
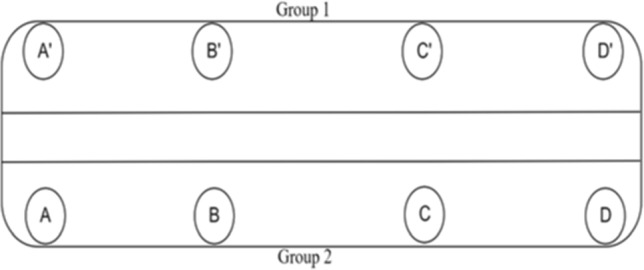
Door locations in the system where the data are collected

The datasets are available on the Cranfield cord repository (Namoano et al., 2020a). Over time, such doors may fail to achieve their goal and show unusual behaviour visible through the profiles. Unusual behaviours include, for example, faster or slower speed increasing or reducing hence the required time to achieve the closing or the opening. These affect the current profile, as the controllers adjust the speed automatically by changing the current. Other key unusual behaviours are the overshoot and the variation of the speed during the steady phase, which is either the manifestation of the defective components of the door or reading sensor issues. The present work seeks to segment and annotate groups of such similar doors, as shown in Fig. 6 where each door represented from the letter A to D may have different utilisation rates. An investigation can be, for instance, looking for unusual deviations between the two groups.

## FEATURES

As already mentioned, feature extraction is critical for the performance of the methods. It enables the capture of system dynamics, which is sensitive to deviations from normalities.

Tables 1 and 2 present, respectively, the features extracted from engines and door profiles. For the doors, the features include overshoot, the rising gradient, the settling time, and the steady state mean and standard deviation. These features are sensitive to fast or slow doors opening or closing, shaking doors due to spalling defects, and unexpected changes. For engines, features are extracted during acceleration, gear change, deceleration, and steady-state periods. Such features are important for detecting sensors and engine faults.

**Table 1:** Features extracted from door profiles

Signal	Motion	Feature
Speed	Opening	Overshoot Steady-state mean Settling time Gradients Steady-state standard deviation
	Closing	Overshoot Steady state mean Settling time Gradients Steady state standard deviation
Current	Opening	Overshoot Steady state mean Settling time Gradients Steady state standard deviation
	Closing	Overshoot Steady state mean Settling time Gradients Steady state standard deviation

**Table 2:** Features extracted from engine datasets

Signal	Motion	Feature
Engine RPM	Acceleration	Overshoot Settling time Gradients Steady state standard deviation
	Gear Change	Change duration Change gradient
	Deceleration	Dips Gradient
Air Pressure Change	Acceleration	Overshoot Settling time Gradients Steady state standard deviation
	Gear Change	Change duration Change gradient
	Deceleration	Dips Gradient
Exhaust Temperature		Unexpected peaks Steady standard deviation

## Algorithmic parameters setting

For effective detection, different parameters setting is applied to each category of the dataset. Table 3 shows the number of multivariate used in each category (*K*), the number of observations in each multivariate time-series (*n*), the window length *w*_*s*_, the penalty (*β*), the sparsity penalty (*λ*), and the ADAMM regularisation parameter (*ρ*) applied to the model. These optimal parameter values are obtained through a grid search algorithm. Due to computational limitations, the parameters search interval was set [start;end;step] as follows: w_s_ = [2;300;1], β = [1–2000,1], λ = [0;1;0.01], ρ = [0;1;0.01].

**Table 3:** Parameters setting used for the model

Datasets	*K*	*n*	*w*_*s*_	$\beta$	$\lambda$	$\rho$
Engine_1	3	120,000	18	325	0.2	0.98
Engine_2	3	120,000	18	350	0.6	0.60
Doors	2	36,000	20	198	0.11	0.80

Table 4 summarises the parameter value obtained by empirical tests in BOCPD methods. For BOCPD, a prior Gaussian distribution assumption is made. The hazard parameter, which is used jointly with the Gaussian distribution for sufficient statistics computation, has also been tested empirically. Grid searches were also applied to the BOCPD methods to select best parameters. The threshold parameter enables the adjustment for the detection sensitivity. Higher thresholds provide higher sensitivity, which can lead to noise detection as anomalies. Hence the thresholds were empirically set to avoid noise present in the data such as the engine E3 Exhaust temperature in Fig. 3, or the subtle signal differences also observed in Fig. 3, across engine charge air temperatures, and pressures. These differences are considered as normal as in normal operation, depending on the leading vehicle, DMU engines have different behaviours, either generating more power or less in comparison to the others engines.

**Table 4:** Parameters settings used in the compared methods

Dataset	BOCPD	Hazard grid search
Engines_1	Gaussian, hazard = 95, threshold = 0.5	[1;10,000;1]
Engines_2	Gaussian, hazard = 145, threshold = 0.5	[1;10,000;1]
Doors	Gaussian, hazard = 25, threshold = 0.001	[1;10,000;1]

Three performance indicators – precision, recall and F1-score, are involved in the assessment of the approaches. Precision measures the percentage of correctly annotated cases, while recall represents the percentage of actual changes that are annotated correctly. The F1-score serves as the test accuracy metric, representing the correctness of all identified cases.

13 $$Precision\left(P\right)= \frac{Number of Correct classification}{Number of annotation}.100$$

14 $$Recall \left(R\right)= \frac{Number of Correct annotations}{Number of true classification}.100 $$

15 $$F1-score\left(F1\right)= 2\frac{P.R}{P+R} $$

## Results and discussion

To demonstrate the effectiveness of the proposed method in detecting anomalies within multivariate time-series, the performance of the proposed G-BOCPD method has been compared against the performance of six time-series analysis methods, obtained from the literature. These methods include 1NN-DTW (Xi et al., 2006), SAX–VSM (Senin & Malinchik, 2013), Shapelet forests (Patri et al., 2014), MC-DCNN (Zheng et al., 2014), FDC-CNN (Lee et al., 2017) and MTS-CNN (Hsu & Liu, 2021), as discussed in Sect. 1. The evaluation employed precision, recall and F1-score metrics, as discussed in (Hsu & Liu, 2021), to assess the performance of the proposed method through sixfold cross-validation. Tables 5, 6 and 7 summarise the performance comparisons for anomaly detection among the seven aforementioned methods in precision, recall and F1-score, employing data from Engine 1, Engine 2, and Doors, respectively.

**Table 5:** Engine 1 anomaly detection results

Methods	Precision	Recall	F1
1NN-DTW (Xi et al., 2006)	0.30	0.20	0.24
SAX–VSM (Senin & Malinchik, 2013)	0.85	0.39	0.53
Shapelet forests (Patri et al., 2014)	0.90	0.77	0.82
MC-DCNN (Zheng et al., 2014)	0.84	0.90	0.86
FDC-CNN (Lee et al., 2017)	0.91	0.94	0.92
MTS-CNN (Hsu & Liu, 2021)	0.90	0.91	0.90
**G-BOCPD**	**0.97**	**0.98**	**0.96**

**Table 6:** Engine 2 anomaly detection results

Methods	Precision	Recall	F1
1NN-DTW (Xi et al., 2006)	0.35	0.25	0.29
SAX–VSM (Senin & Malinchik, 2013)	0.84	0.42	0.56
Shapelet forests (Patri et al., 2014)	0.84	0.80	0.82
MC-DCNN (Zheng et al., 2014)	0.64	0.80	0.71
FDC-CNN (Lee et al., 2017)	0.89	0.78	0.83
MTS-CNN (Hsu & Liu, 2021)	0.91	0.93	0.91
**G-BOCPD**	**0.98**	**0.97**	**0.97**

Following the anomaly detection results of Engine 1 in Table 5, it can be seen that Shapelet Forests, FDC-CNN, MTS-CNN and G-BOCPD provide high precision with average values equal to or higher than 0.9. Additionally, it is observed that although the average precision results of SAX–VSM and MC-DCNN are very close (~ 0.845), the average recall of SAX–VSM is significantly lower than MC-DCNN by 51%. In terms of the average precision and recall, MC-DCNN, FDC-CNN, MTS-CNN and the proposed G-BOCPD provide better results than 1NN-DTW, SAX–VSM, and Shapelet Forests. Overall, considering the results of Engine 1, the proposed G-BOCPD outperforms the other six methods with high average precision (0.96), recall (0.98) and F1 (0.97). It is also observed that 1NN-DTW would be the least preferred method as the values of average precision, recall and F1-score are the lowest among the other methods. It is worth mentioning that similar observations were made for the results obtained from the analysis of Engine 2 (Table 5) and Doors (Table 6) datasets.

**Table 7:** Doors anomaly detection results

Methods	Precision	Recall	F1
1NN-DTW (Xi et al., 2006)	0.61	0.42	0.49
SAX–VSM (Senin & Malinchik, 2013)	0.81	0.60	0.69
Shapelet forests (Patri et al., 2014)	0.84	0.90	0.87
MC-DCNN (Zheng et al., 2014)	0.84	**0.96**	0.90
FDC-CNN (Lee et al., 2017)	0.86	0.94	0.90
MTS-CNN (Hsu & Liu, 2021)	0.90	0.92	**0.91**
**G-BOCPD**	**0.94**	0.88	**0.91**

Moreover, in terms of the anomaly detection results of Engine 2 in Table , it is seen that only MTS-CNN and the proposed G-BOCPD have an average precision above 0.9. Moreover, in terms of the average precision and recall, Shapelet Forests, FDC-CNN, MTS-CNN and G-BOCPD provide better performance compared to 1NN-DTW, SAX–VSM and MC-DCNN. Overall, the anomaly detection results for Engine 2 demonstrate that the proposed G-BOCPD outperforms the other six methods, achieving high average precision (0.98), recall (0.97) and F1-score (0.97), consistent with the observations from the results of Engine 1.

Additionally, similar to Engine 2, the results of Doors in Table 5 show that only MTS-CNN and the proposed G-BOCPD provide highly precise results, equal to or higher than 0.9. It is interestingly observed in Table 5 that the proposed G-BOCPD outperforms the other six methods with high average precision (0.94) and F1-score (0.91). It is also noticed that MTS-CNN and the proposed G-BOCPD have the highest F1-score values, equal to 0.91, compared to the other methods. However, the average recall of the proposed G-BOCPD is 0.88, lower than the recall values of Shapelet Forests, MC-DCNN, FDC-CNN and MTS-CNN. The highest average recall value is obtained from MC-DCNN and equals 0.96. In conclusion, G-BOCPD-based tests reveal that the average precision, recall and F1-score values are the highest for the datasets of Engine 1 and Engine 2, being consistently above 95%, compared to the Doors dataset in which a lower F1-score can be seen (88%). One of the main reasons for this lower recall value for the Doors dataset is that the proposed G-BOCPD poorly segments the data, leading to inefficiency in the clustering. This limitation will be further explored in future research.

## Conclusion

This paper has proposed a novel graphical model-based annotation method, called G-BOCPD, for automatically detecting anomalies in multi-channel multivariate time-series data. The novelty of G-BOCPD lies in the hybridisation of the graphical lasso and expectation maximisation algorithms that enable capturing both internal and external dependencies within the data. This hybrid approach, which addresses the challenge of detecting anomalies by considering the complex relationships between variables, identifies segments in multivariate time-series that exhibit different behaviour or patterns. These segments are then annotated to highlight the variations. The proposed method alternates between estimating the concentration matrix, which represents the dependencies between variables, using the graphical lasso algorithm, and annotating the segments using a minimal path clustering method that allows for a comprehensive understanding of variations within the data.

Two real-world engineering datasets have been used to validate the G-BOCPD method. The first dataset focused on DMU train engines with faulty behavioural patterns while the second dataset involved train doors’ data with different utilisation rates that resulted in varying degradation stages. To ensure a comprehensive representation of system dynamics, relevant features such as overshoot, gradients, and steady-state standard deviations were extracted from both datasets. These features provided valuable information about the behaviour and characteristics of the systems under observation. The application of the G-BOCPD method to these case studies demonstrated its practicality and effectiveness in detecting anomalies in diverse scenarios within the engineering domain. By leveraging the combination of graphical modelling techniques and clustering methods, the G-BOCPD method proved capable of automatically capturing complex dependencies and variations within the time-series data.

Quantitative experiments have been conducted to compare the performance of G-BOCPD with alternative time-series methods in terms of precision, recall, and F1-score. The results obtained through sixfold cross-validation on three datasets (i.e. Engine 1, Engine 2, and Doors) have shown that G-BOCPD achieved competitive results, indicating its effectiveness in anomaly detection compared to existing approaches in the field. Across the three datasets, G-BOCPD consistently achieved high average precision (0.96–0.98), recall (0.97), and F1-score (0.96–0.97). It outperformed other methods in terms of average precision and recall, with 1NN-DTW being the least preferred method. G-BOCPD and MTS-CNN provided highly precise results for the Doors dataset, but G-BOCPD had a lower average recall. Overall, G-BOCPD consistently achieved precision, recall, and F1-score values above 95% for Engine 1 and Engine 2 datasets. The Doors dataset showed a lower F1-score (88%) due to inefficient data segmentation by G-BOCPD, which adversely affected recall. Further research will address this limitation.

Further investigations can be undertaken to enhance the G-BOCPD method. A potential future direction could be to explore methods for estimating the number of clusters. The current approach assumes prior knowledge of the number of clusters, which may not always be available. Therefore, additional research could explore methods for automatically estimating the number of clusters by considering a penalty for each new cluster utilised. This would enable the method to handle cases where the number of clusters is unknown, improving its flexibility and adaptability.

## Data Availability

The data that support the findings of this study are available from the corresponding author upon request.

## Footnotes

1 A set of single channels with univariate or multivariate input time-series data with different features or variables. Each channel has a distinct distribution of values.

2 A set of univariate time-series data with more than one time-dependent features or variables. Each feature depends not only on its past values but also has some dependency on other features. Using this dependency, future values can be predicted.

3 The statistical properties of time-series change over time.

4 Unwanted changes to signals during their capture, storage, transmission, processing, or conversion.
